# A model for predicting secondary traumatic stress in emergency nurses: the roles of occupational stress, empathy, and psychological resilience

**DOI:** 10.3389/fmed.2025.1712821

**Published:** 2025-12-18

**Authors:** Aiping Dong, Yuefang Pan, Fanghua He

**Affiliations:** Emergency Department, Hangzhou Linping District First People's Hospital, Hangzhou, Zhejiang, China

**Keywords:** ED nurses, empathy, nursing stress, predictive model, psychological resilience, secondary traumatic stress

## Abstract

**Background:**

The high-stress environment of the emergency department (ED) predisposes nurses to significant occupational stress, which can precipitate compassion fatigue and secondary traumatic stress (STS). STS adversely affects nurses’ wellbeing and may compromise both their professional performance and patient care quality. Investigating the predictive roles of nursing stress, empathy, and psychological resilience on STS in ED nurses, and constructing a reliable predictive model, is therefore clinically imperative.

**Objective:**

This study aimed to develop a predictive model for STS among ED nurses based on nursing stress, empathy, and psychological resilience, intending to inform psychological support strategies and management practices.

**Methods:**

A cross-sectional survey was conducted using convenience sampling to recruit 186 ED nurses from a single hospital. Data were collected via questionnaires, including the Vicarious Traumatization Questionnaire (VTQ), the Chinese version of the Interpersonal Reactivity Index (IRI-C), the Perceived Stress Scale (PSS), and the Connor-Davidson Resilience Scale (CD-RISC). Data analysis involved descriptive statistics, correlation analysis, and multiple linear regression to build a multidimensional model of factors influencing STS.

**Results:**

The results indicated that nursing stress, empathy, and psychological resilience were significantly associated with STS. Higher levels of nursing stress and empathy, along with lower levels of psychological resilience, were positively correlated with higher STS scores. Sub-factors such as personal distress, emotional resonance, and exposure to traumatic events had the strongest associations. The regression model, incorporating these predictors, explained 56.00% of the variance in STS scores.

**Conclusion:**

STS in ED nurses is influenced by multiple factors, including nursing stress, empathy, and psychological resilience, with resilience serving as an important protective factor. The developed regression model provides valuable insights into these associations and holds clinical utility in guiding interventions aimed at enhancing nurse mental health, refining the work environment, and improving patient care quality. These findings suggest the need for targeted psychological support strategies in ED settings.

## 1 Introduction

As a crucial part of the clinical nursing team, emergency department (ED) nurses are tasked with handling high-risk and critically ill patients. The emergency environment is characterized by high dynamism, urgency, and uncertainty, requiring nurses to make rapid judgments and decisions on complex cases within limited time frames, while frequently encountering life-and-death situations of patients. This exposes them to significant psychological pressure. Prolonged exposure to such high-stress environments makes ED nurses prone to occupational psychological distress, particularly secondary traumatic stress (STS) (1, 2). STS is an emotional and psychological state related to the trauma experiences of others, manifested as an excessive empathic response to others’ suffering, leading to emotional exhaustion, cognitive impairment, and significant damage to physical and mental health (3, 4). For ED nurses, STS not only affects individual mental health but can also lead to burnout, occupational dysfunction, and decreased quality of patient care. In severe cases, it may impact the overall effectiveness of the nursing team (5–7). Therefore, identifying and predicting the risk factors for STS is crucial for developing accurate psychological intervention strategies and optimizing ED nursing management. Existing STS prediction models are mostly limited to specific nursing fields, lacking an in-depth understanding of the unique characteristics of ED nurses’ work and rarely considering the moderating role of individual psychological traits in STS. Hence, this study aims to construct a predictive model for STS in ED nurses by quantifying the three key variables: nursing stress, empathy, and psychological resilience. Through this study, we can identify the critical factors influencing STS in ED nurses, provide scientific evidence for early identification of high-risk nursing groups, develop personalized psychological interventions, and offer theoretical support for optimizing the ED nursing environment and enhancing nurses’ mental health.

## 2 Objects and methods

### 2.1 Research subjects

This study adopts a cross-sectional design and uses a convenient sampling method to select 186 emergency department nurses from our hospital as research subjects. The inclusion criteria are as follows: (1) Registered nurses working in the emergency department; (2) Engaged in emergency nursing for ≥1 year; (3) Voluntarily participate in the study and sign an informed consent form. The exclusion criteria are: (1) History of severe mental or psychological disorders such as major depression or anxiety disorders; (2) Experienced major life events (e.g., bereavement, serious illness) in the past 3 months; (3) Interns or nurses undergoing further education.

To ensure the adequacy of the sample size for the regression analysis, we refer to the widely accepted rule of 10–15 cases per predictor. This guideline suggests that for each predictor variable included in a regression model, the sample size should be at least 10 to 15 times the number of predictors to achieve reliable results and avoid overfitting. In this study, there are 5 predictors included in the regression model (e.g., empathy subdimensions, personal distress, traumatic events). With a sample size of *n* = 186, we are well above the required threshold (10 × 5 = 50, and 15 × 5 = 75 cases). Therefore, the sample size is deemed adequate to support the regression analysis and provide statistically reliable results. This ensures that the model has sufficient statistical power to detect significant relationships without the risk of overfitting or underfitting the data. The study was approved by the Lunshan Review Committee of Linping First Hospital, with approval number 2023 Lunshen 063.

### 2.2 Survey tools

**Figure 1:**
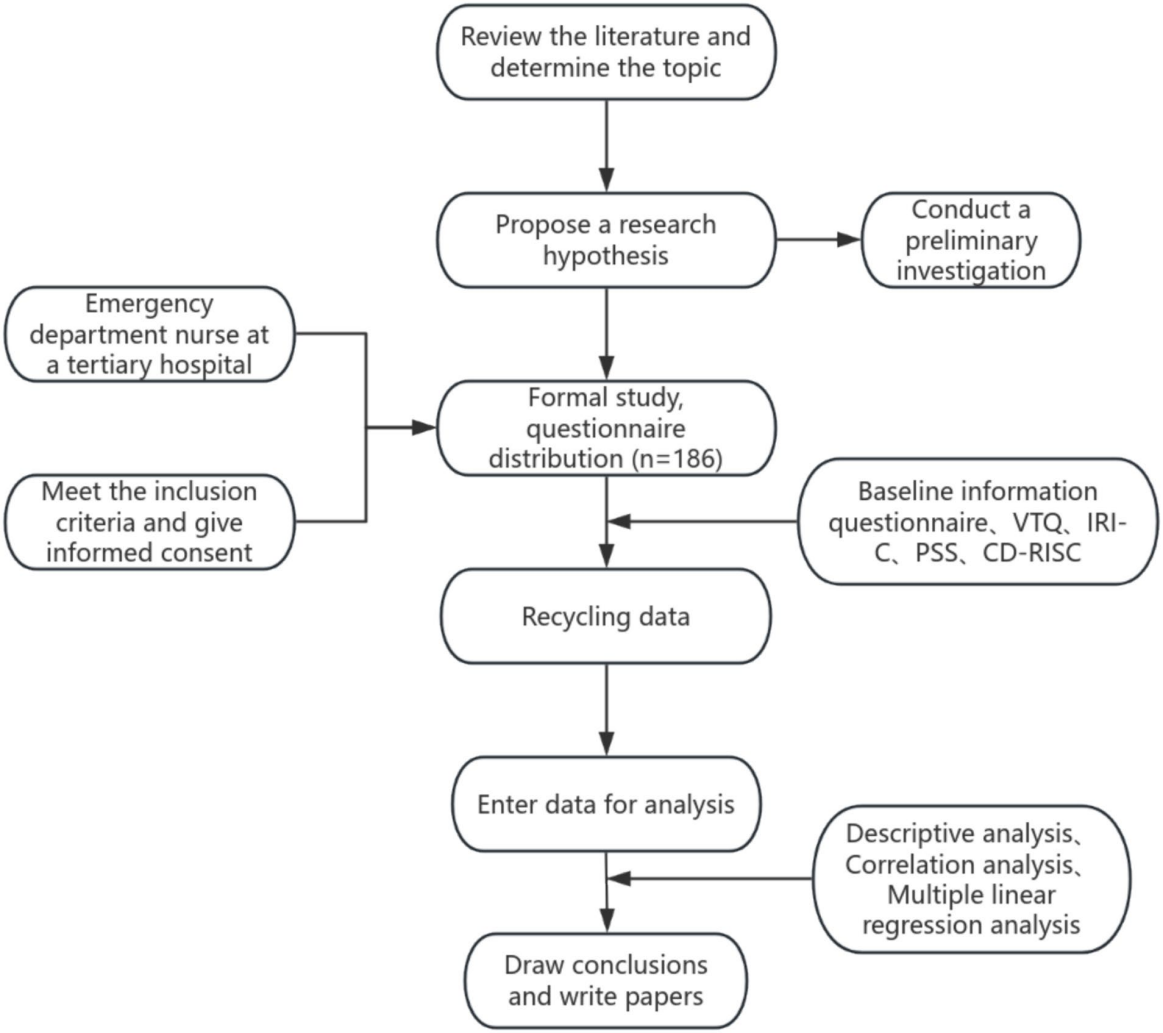
Flowchart detailing the research process: Starting with reviewing literature and determining the topic, formulating a hypothesis, conducting a preliminary investigation, and selecting participants (emergency department nurses). The process includes obtaining informed consent, distributing study questionnaires (n=186), and performing data analysis (descriptive statistics, correlation, and regression analysis) to draw conclusions.

This study aims to construct a predictive model of STS in emergency department nurses based on factors such as nursing stress, empathy, and psychological resilience, using standardized questionnaires for data collection. To ensure the scientific rigor and reliability of the data, several validated scales have been selected, which have been widely used in related fields and have high reliability and validity. The selection of survey tools adheres to the principles of professionalism, effectiveness, and operability. The surveys will be distributed to emergency department nurses in the form of self-administered questionnaires, and the data will be collected either electronically or on paper. The research team will ensure the anonymity and confidentiality of all information and strictly check all questionnaires and data entry. The research roadmap is shown in Figure 1.

#### 2.2.1 Baseline data questionnaire

Based on a review of relevant literature and the objectives and content of this study, a general demographic data questionnaire was self-designed for emergency department nurses, considering their actual situation. The questionnaire includes: age, gender, marital status, years of work experience, highest level of education, employment type, professional title, personality type, monthly income, traumatic events, psychological trauma training, and career re-selection. The design of this questionnaire aims to collect basic demographic information of the nurses, so that possible confounding variables can be controlled in subsequent data analysis and provide foundational data for constructing the predictive model of STS.

Work experience was coded into three categories: ‘1–3 years’, ‘4–5 years’, and ‘6–10 years’, with ‘1–3 years’ serving as the reference group. This selection was based on literature (8, 9) indicating that nurses with 1–3 years of experience are typically in a transitional phase of their careers, facing higher occupational stress while gradually developing effective coping mechanisms. Therefore, using this group as a benchmark helps compare stress responses among nurses with different experience levels. Furthermore, the 4–5 year and 6–10 year cutoffs were chosen to represent different stages of experience in a nurse’s career, reflecting different processes of adaptation and coping with work stress. This selection of cutoffs aligns with existing research standards for examining differences in stress and psychological resilience across different experience levels.

#### 2.2.2 Vicarious traumatization questionnaire (VTQ)

The Vicarious Traumatization Questionnaire (VTQ) (10, 11) is a specialized tool designed to assess STS in individuals working in high-pressure environments, such as disaster relief workers, psychotherapists, and emergency care professionals (e.g., emergency department nurses). This questionnaire measures the emotional, cognitive, and behavioral changes that occur after exposure to others’ traumatic experiences, helping to identify and quantify the extent of STS. The scale includes five dimensions: emotional responses, cognitive changes, behavioral changes, physiological responses, and life beliefs, with a total of 38 items. It employs a Likert 5-point scale (1 = Never, 2 = Occasionally, 3 = Sometimes, 4 = Often, 5 = Always) for responses. The total score range is 38–190, with higher scores indicating greater severity of trauma. The VTQ has been validated in disaster relief settings, showing strong reliability and validity. In this study, the Cronbach’s alpha coefficient for the scale was 0.934, indicating excellent internal consistency.

#### 2.2.3 Chinese version of the interpersonal reactivity index

The Interpersonal Reactivity Index (IRI) is a psychological tool used to assess an individual’s empathy and emotional responses in interpersonal contexts. Developed by Davis in 1980, the IRI (12) has been widely applied in psychology, sociology, and clinical research. The Chinese version of the Interpersonal Reactivity Index (IRI-C) (13) was adapted and culturally validated for use with the Chinese population, designed to assess empathy and emotional responsiveness, particularly in social interactions. The IRI-C includes four dimensions: Empathic Concern (EC), Fantasy (FS), Personal Distress (PD), and Perspective Taking (PT). Each dimension consists of a set of items, and the responses are quantified to assess various aspects of empathy. The total score range is 0–88 points, with higher scores reflecting higher levels of empathy. In this study, the Cronbach’s alpha coefficient for the IRI-C was 0.852, indicating good internal consistency.

#### 2.2.4 Perceived stress scale

The Perceived Stress Scale (PSS) (14), developed by Cohen et al. in 1983, is a widely used tool to assess an individual’s perceived stress level over the past month. The scale employs a self-report method to measure subjective stress experiences across various life situations. It is commonly used in psychology, health research, and clinical stress assessments, particularly for healthcare workers. The PSS aims to capture the intensity of perceived stress and the sense of control, evaluating how situational factors and emotional responses impact mental health. The Chinese version of the PSS (PSS-C) (15) has been localized and validated for use with Chinese nurses, demonstrating good reliability and validity. The PSS-C includes three dimensions: perceived stress, sense of control, and emotional response, with a total of 10 items. Each item is rated on a 0–4 scale, and the total score ranges from 0 to 40, with higher scores indicating greater perceived stress and a lower sense of control. In this study, the Cronbach’s alpha coefficient for the PSS-C was 0.855, indicating good internal consistency.

#### 2.2.5 Connor-Davidson resilience scale

The Connor-Davidson Resilience Scale (CD-RISC) (14), developed by psychologists Jonathan R. Connor and Kathryn M. Davidson in 2003, is designed to assess an individual’s ability to adapt to adversity, stress, or trauma, reflecting their psychological resilience. This scale is widely used in clinical psychology, health psychology, and sociology to measure an individual’s coping abilities, mental health, emotional regulation, and the effectiveness of coping strategies. The Chinese version of the CD-RISC (15) has been adapted for local context and validated in multiple studies, demonstrating strong reliability and validity. It is widely used for resilience assessments in Chinese populations, particularly in clinical interventions, mental health promotion, and health-related interventions. The original CD-RISC consists of 25 items, focusing on traits related to adaptation, emotional regulation, problem-solving ability, and self-efficacy in facing adversity. The Chinese version retains the original structure while adjusting expressions to align with Chinese cultural context. It includes four dimensions: personal confidence and self-efficacy, emotional regulation and self-control, interpersonal relationships and social support, and cognitive and problem-solving ability, with a total of 25 items. The total score ranges from 0 to 100 points, with higher scores indicating greater psychological resilience and better adaptability to life’s challenges. In this study, the Cronbach’s alpha coefficient for the CD-RISC was 0.932, indicating excellent internal consistency.

### 2.3 Statistical analysis

This study used SPSS 27.0 software for data analysis. Quantitative data, after normality testing, were expressed as mean ± standard deviation (M ± SD) if they conformed to a normal distribution and had homogeneity of variance; otherwise, the median (Q25, Q75) was used. Categorical data were expressed as frequency, percentage (%), and proportion (%). In the difference tests, independent samples t-tests were used to compare differences between two groups, and one-way ANOVA was used to compare differences among multiple groups. In the correlation analysis, Pearson correlation analysis was used to analyze the correlation between variables such as vicarious trauma, empathy, and psychological resilience. In the regression analysis, stepwise regression was used to screen significant predictor variables. The relationship between variables and STS was assessed by calculating the regression coefficient (B), standard error (SE), standardized coefficient (*β*), confidence interval (CIs), and *p*-value for each predictor variable. We also performed tests for multicollinearity (VIF) and residual analysis to ensure the stability and validity of the model. All statistical tests were two-tailed probability tests, and the difference was considered statistically significant when *p* < 0.05.

## 3 Results

### 3.1 Baseline data analysis of emergency department nurses

The subjects of this study were predominantly female, with ages mainly ranging from 25 to 45 years. Most nurses had 1 to 10 years of work experience, and the proportion of married nurses was significantly higher than that of unmarried and divorced nurses. The majority of the nurses were employed under contract, with a bachelor’s degree being the most common highest level of education, and the majority held the titles of “Nurse” or “Head Nurse.” The monthly income for most nurses fell within the range of 7,001–9,000 RMB. In terms of personality type, the nurses displayed characteristics of an intermediate personality between extroversion and introversion. The majority of nurses had not received training in psychological trauma. Furthermore, the re-selection rate for the nursing profession among emergency department nurses was low, indicating a relatively high job stability. The baseline data analysis is summarized in Table 1.

**Table 1:** Baseline data analysis.

Variable	Group	Number	Composition ratio (%)
Age (years)	<25	53	28.50
	25–30	49	26.20
	31–40	62	33.10
	41–50	20	10.75
	>50	2	1.45
Gender	Male	7	3.76
	Female	179	96.24
Marital Status	Unmarried	72	38.50
	Married	107	57.70
	Divorced	7	3.80
Work experience (years)	1–3	66	35.40
	4–5	34	18.20
	6–10	41	21.80
	11–15	25	13.30
	>16	20	11.30
Highest education	Bachelor’s	173	93.10
	Master’s or above	13	6.90
Employment type	Regular Staff	40	21.50
	Contract Staff	136	73.10
	Other	10	5.40
Title	Nurse	66	35.40
	Nurse practitioner	50	26.90
	Head nurse	63	33.80
	Deputy chief nurse	7	3.90
Personality type	Introverted	36	19.20
	Extroverted	29	15.40
	Intermediate	121	65.40
Monthly income (RMB)	≤3,000	16	8.40
	3,001–5,000	34	18.50
	5,001–7,000	38	20.80
	7,001–9,000	59	31.50
	≥9,001	39	20.80
Traumatic event	Yes	43	23.10
	No	143	76.90
Psychological trauma training	Yes	31	16.90
	No	155	83.10
Career re-selection	Yes	63	33.80
	No	123	66.20

### 3.2 General description of emergency department nurses’ STS, empathy, nursing stress, and psychological resilience

#### 3.2.1 Overall situation of STS in emergency department nurses

In this study, the total score for STS in emergency department nurses was 86.97 ± 23.78, with an item average score of 2.27 ± 0.61. There were 21 nurses with total scores greater than the theoretical median, resulting in a STS occurrence rate of 11.29%. In the five dimensions of cognitive, emotional, behavioral, life beliefs, and physiological responses, the number of nurses scoring above the theoretical median were as follows: cognitive responses (10 nurses, 5.38%), emotional responses (28 nurses, 15.05%), behavioral responses (31 nurses, 16.67%), life beliefs (36 nurses, 19.35%), and physiological responses (30 nurses, 16.13%). The data indicate that emergency department nurses exhibit relatively high levels of STS in emotional and behavioral responses, with some nurses showing notable traumatic symptoms across multiple dimensions, suggesting the widespread and severe nature of STS in this population. The details are shown in Table 2.

**Table 2:** Overall situation of secondary traumatic stress in emergency department nurses.

Item	Number of items	Total score (Mean ± SD)	Mean score per item (±SD)
Secondary traumatization	38	86.97 ± 23.78	2.27 ± 0.61
Cognitive response	5	8.51 ± 3.27	1.69 ± 0.64
Emotional response	9	22.06 ± 6.88	2.44 ± 0.75
Behavioral Response	7	16.44 ± 4.72	2.33 ± 0.66
Life beliefs	6	15.64 ± 4.73	2.59 ± 0.78
Physiological response	11	24.29 ± 8.65	2.19 ± 0.77

#### 3.2.2 Overall situation of empathy in emergency department nurses

In this study, the total empathy score for emergency department nurses was 55.11 ± 13.55, with an average score of 2.50 ± 0.61. A total of 142 nurses (76.34%) scored above the theoretical median. In the four dimensions of perspective taking, imaginative empathy, empathetic concern, and personal distress, the number of nurses scoring above the theoretical median were as follows: perspective taking (120 nurses, 64.52%), imaginative empathy (125 nurses, 67.20%), emotional resonance (139 nurses, 74.73%), and personal distress (68 nurses, 36.56%). The data indicate that most emergency department nurses perform well in empathy, especially in empathetic concern. However, a certain proportion of nurses scored below the theoretical median in personal distress, suggesting that some nurses may experience difficulties with emotional regulation and self-protection when faced with patient suffering (Table 3).

**Table 3:** Overall situation of empathy in emergency department nurses.

Item	Number of Items	Total Score	Average Score per Item
Empathy	22	55.11 ± 13.55	2.50 ± 0.61
Perspective taking	5	12.44 ± 3.64	2.48 ± 0.72
Imaginative empathy	6	15.02 ± 4.17	2.49 ± 0.69
Emotional resonance	6	17.51 ± 4.10	2.91 ± 0.67
Personal distress	5	10.13 ± 4.51	2.02 ± 0.89

#### 3.2.3 Overall situation of nursing stress in emergency department nurses

In this study, the total nursing stress score for emergency department nurses was 31.57 ± 4.93, with an average score of 3.16 ± 0.49. A total of 120 nurses (64.52%) scored above the theoretical median. In the three dimensions of stress perception, sense of control, and emotional response, the number of nurses scoring above the theoretical median were as follows: stress perception (98 nurses, 52.69%), sense of control (102 nurses, 54.84%), and emotional response (88 nurses, 47.31%). The data suggest that most emergency department nurses experience significant levels of stress in terms of both stress perception and sense of control. In particular, a number of nurses displayed strong emotional responses, indicating significant challenges in emotional regulation under high-pressure conditions. The findings highlight that emergency department nurses face considerable stress, particularly in emotional responses and sense of control, which may affect their work efficiency and mental health. Appropriate stress management and emotional support interventions are necessary to reduce their stress burden (Table 4).

**Table 4:** Overall situation of nursing stress in emergency department nurses.

Item	Number of items	Total score	Average score per item
Nursing stress	10	31.57 ± 4.93	3.16 ± 0.49
Stress perception	5	14.42 ± 2.88	2.88 ± 0.58
Sense of control	4	11.73 ± 2.50	2.93 ± 0.62
Emotional response	1	2.85 ± 0.42	2.85 ± 0.42

#### 3.2.4 Overall situation of psychological resilience in emergency department nurses

In this study, the total psychological resilience score for emergency department nurses was 71.36 ± 8.24, with an average score of 2.85 ± 0.33. A total of 138 nurses (74.19%) scored above the theoretical median. In the four dimensions of personal confidence and self-efficacy, emotional regulation and self-control, interpersonal relationships and social support, and cognitive and problem-solving abilities, the number of nurses scoring above the theoretical median were as follows: personal confidence and self-efficacy (115 nurses, 61.83%), emotional regulation and self-control (112 nurses, 60.22%), interpersonal relationships and social support (98 nurses, 52.69%), and cognitive and problem-solving abilities (121 nurses, 65.05%). The data indicate that most emergency department nurses exhibit strong psychological resilience, particularly in the areas of personal confidence, self-efficacy, and cognitive problem-solving abilities. However, a certain proportion of nurses scored below the theoretical median in the dimension of interpersonal relationships and social support, suggesting that some nurses may face difficulties in obtaining social support and managing interpersonal relationships, which could impact their overall psychological resilience (Table 5).

**Table 5:** Overall situation of psychological resilience in emergency department nurses.

Item	Number of items	Total score	Average score per item
Psychological resilience	25	71.36 ± 8.24	2.85 ± 0.33
Personal confidence and self-efficacy	6	17.83 ± 3.10	2.97 ± 0.52
Emotional regulation and self-control	7	19.47 ± 4.13	2.78 ± 0.59
Interpersonal relations and social support	6	16.29 ± 3.65	2.72 ± 0.61
Cognitive and problem-solving abilities	6	17.77 ± 3.45	2.96 ± 0.58

### 3.3 Analysis of differences in STS, empathy, nursing stress, and psychological resilience based on demographic variables

The influence of various demographic variables on the scores for STS, empathy, nursing stress, and psychological resilience among emergency department nurses is shown in Table 6. The scores for these variables followed a normal distribution across all demographic groups. For the variables of gender, experience with traumatic events, psychological trauma training, and career re-selection, an independent samples t-test was used. For the variables of age, years of work experience, marital status, employment type, highest education level, professional title, monthly income, and personality type, one-way ANOVA was employed.

**Table 6:** Comparison of scores across different demographic variables for emergency department nurses.

Variable	Group	Secondary traumatic stress	Empathy	Nursing stress	Psychological resilience
Age (years)	<25	84.80 ± 21.24	61.77 ± 12.60	34.20 ± 3.56	74.20 ± 6.80
	25–30	91.52 ± 27.47	60.05 ± 13.12	32.15 ± 4.12	76.50 ± 7.50
	31–40	86.76 ± 24.62	50.59 ± 7.71^c^	33.65 ± 2.98	78.13 ± 7.20
	41–50	83.49 ± 19.62	56.00 ± 12.65	35.40 ± 3.12	78.30 ± 6.90
	>50	78.99 ± 14.13	45.00 ± 7.06^c^	37.25 ± 2.45	75.50 ± 7.30
Gender	Male	76.19 ± 24.61*	53.03 ± 11.76	36.85 ± 3.32	74.67 ± 7.50
	Female	89.54 ± 22.97	57.63 ± 12.20	31.50 ± 3.04	79.50 ± 6.70
Marital status	Unmarried	85.34 ± 23.15	60.28 ± 12.33	34.45 ± 3.24	77.05 ± 7.21
	Married	88.68 ± 24.87	53.47 ± 11.01^e^	32.75 ± 2.80	78.36 ± 7.50
	Divorced	86.39 ± 20.47	52.79 ± 14.93	33.90 ± 3.06	76.30 ± 7.30
Work experience (years)	1–3	85.15 ± 22.12^a^	62.30 ± 12.41	34.10 ± 2.60	78.50 ± 6.80
	4–5	95.49 ± 26.60	52.91 ± 12.77^d^	31.85 ± 3.34	79.48 ± 6.70
	6–10	80.27 ± 25.37^a^	52.60 ± 9.60^d^	36.50 ± 2.90	77.50 ± 7.20
	11–15	101.93 ± 29.42	55.40 ± 12.04^d^	30.40 ± 2.10	76.00 ± 7.00
	>16	81.79 ± 15.98^a^	49.59 ± 7.08^d^	38.25 ± 1.70	80.11 ± 6.80
Highest education	Bachelor’s	86.87 ± 23.41	55.98 ± 12.09	34.00 ± 3.10	76.50 ± 7.30
	Master’s or above	89.62 ± 31.78	67.49 ± 5.71	31.50 ± 2.15	80.00 ± 7.10
Employment type	Regular Staff	90.24 ± 21.86	54.88 ± 11.56	32.80 ± 2.90	77.07 ± 7.50
	Contract Staff	85.61 ± 24.87	56.44 ± 12.07	35.50 ± 3.50	75.33 ± 7.30
	Other	92.42 ± 31.42	68.28 ± 12.28	30.70 ± 2.20	80.50 ± 7.20
Title	Nurse	86.23 ± 23.70	61.60 ± 12.56	34.25 ± 3.60	76.80 ± 7.40
	Nurse Practitioner	86.90 ± 25.92	57.12 ± 12.42	34.75 ± 2.90	77.30 ± 7.20
	Head Nurse	87.63 ± 23.02	51.38 ± 9.22	36.50 ± 2.30	75.52 ± 7.10
	Deputy Chief Nurse	88.59 ± 22.20	56.79 ± 14.86	33.50 ± 2.80	78.14 ± 7.30
Personality type	Introverted	102.63 ± 28.68^b^	53.29 ± 9.32	30.80 ± 2.60	80.22 ± 6.50
	Extroverted	74.19 ± 20.23	60.55 ± 11.52	38.00 ± 1.50	72.37 ± 7.40
	Intermediate	85.38 ± 20.65^b^	56.44 ± 12.81	35.25 ± 2.85^f^	75.50 ± 7.10
Monthly income (RMB)	≤3,000	88.44 ± 25.43	64.08 ± 15.70	32.40 ± 3.10	76.81 ± 7.30
	3,001–5,000	88.45 ± 22.04	58.32 ± 11.76	33.15 ± 3.20	77.06 ± 7.49
	5,001–7,000	88.18 ± 28.58	58.55 ± 12.96	33.70 ± 2.90	77.51 ± 7.13
	7,001–9,000	87.26 ± 23.06	54.23 ± 10.01	34.10 ± 8.10	76.50 ± 7.21
	≥9,001	83.43 ± 21.79	54.36 ± 12.53	36.50 ± 2.40	74.35 ± 7.14
Traumatic event	Yes	95.62 ± 27.91*	60.09 ± 13.65	30.20 ± 2.30	79.50 ± 6.87
	No	84.38 ± 21.91	55.74 ± 11.63	35.60 ± 3.10	75.23 ± 7.32
Psychological trauma training	Yes	77.78 ± 22.78*	56.41 ± 10.47	31.10 ± 3.20	72.05 ± 7.50
	No	89.06 ± 23.61	56.82 ± 12.62	33.00 ± 2.80	78.13 ± 7.20
Career re-selection	Yes	74.72 ± 18.78*	56.72 ± 11.82	37.00 ± 1.50	70.50 ± 5.32
	No	93.25 ± 23.71	56.76 ± 12.48	32.10 ± 3.00	71.20 ± 7.10

a indicates *P* < 0.05 (compared with the 11–15 years group); b indicates *p* < 0.05 (compared with the introverted group); c indicates *p* < 0.05 (compared with the under 25 years group); d indicates *p* < 0.05 (compared with the 1–3 years group); e indicates *p* < 0.05 (compared with the unmarried group); f indicates *p* < 0.05 (compared with the extroverted group).

Results from the independent samples t-test indicated significant differences (*p* < 0.05) in STS, empathy, nursing stress, and psychological resilience scores based on gender, experience with traumatic events, psychological trauma training, and career re-selection. Specifically, female nurses scored higher in empathy and psychological resilience, while male nurses scored lower in STS. Nurses who had experienced traumatic events had higher STS scores, whereas nurses who had not experienced trauma had higher scores in nursing stress and psychological resilience. Nurses who had received psychological trauma training had lower STS scores, while those who had not received training scored higher in psychological resilience. Nurses with career re-selection intentions scored lower in STS.

Results from the one-way ANOVA revealed significant differences (*p* < 0.05) in STS, empathy, nursing stress, and psychological resilience scores based on age, work experience, education, professional title, and personality type. Specifically, nurses with higher education levels scored higher in empathy and psychological resilience, while those with more years of work experience scored higher in nursing stress and psychological resilience. Introverted nurses had higher STS scores, while extroverted nurses scored higher in empathy.

### 3.4 Relationship analysis of secondary trauma, empathy, nursing stress, and psychological resilience in emergency department nurses

#### 3.4.1 Relationship analysis of secondary trauma and empathy in emergency department nurses

There is a significant positive correlation between the total score of secondary trauma and the total score of empathy (*r* = 0.247, *p* < 0.01). Specifically, in each dimension of empathy, secondary trauma is significantly positively correlated with “perspective-taking” (*r* = 0.332, *p* < 0.01), “fantasy empathy” (*r* = 0.450, *p* < 0.01), and “personal distress” (*r* = 0.488, *p* < 0.01), while it shows a significant negative correlation with “emotional resonance” (*r* = −0.219, *p* < 0.05). This suggests that emergency department nurses with higher secondary trauma exhibit stronger empathy in perspective-taking, fantasy empathy, and personal distress, but weaker empathy in emotional resonance. In addition, the correlation between the various dimensions of secondary trauma and empathy also shows varying degrees of significance. The specific analysis is as follows: In the “cognitive response” dimension, secondary trauma is significantly positively correlated with the total score of empathy (*r* = 0.297, *p* < 0.01), perspective-taking (*r* = 0.327, *p* < 0.01), fantasy empathy (*r* = 0.441, *p* < 0.01), and personal distress (*r* = 0.442, *p* < 0.01), indicating that cognitive response is strongly associated with all dimensions of empathy. In the “emotional response” dimension, the correlation between secondary trauma and empathy is more significant, especially with perspective-taking (*r* = 0.265, *p* < 0.01) and fantasy empathy (*r* = 0.394, *p* < 0.01), which are positively correlated. In the “behavioral response” dimension, secondary trauma is also significantly positively correlated with all dimensions of empathy, particularly with fantasy empathy (*r* = 0.369, *p* < 0.01) and personal distress (*r* = 0.526, *p* < 0.01). In the “life beliefs” dimension, secondary trauma is significantly positively correlated with perspective-taking (*r* = 0.297, *p* < 0.01) and fantasy empathy (*r* = 0.356, *p* < 0.01), but shows no significant correlation with personal distress (*r* = −0.170, *p* > 0.05). In the “physiological response” dimension, secondary trauma is significantly positively correlated with perspective-taking (*r* = 0.266, *p* < 0.01), fantasy empathy (*r* = 0.360, *p* < 0.01), and personal distress (*r* = 0.386, *p* < 0.01), while showing a negative correlation with emotional resonance (*r* = −0.243, *p* < 0.01). See Table 7.

**Table 7:** Correlation analysis of secondary trauma and empathy in emergency department nurses (*r*-values).

Variable	Total empathy score	Perspective-taking	Fantasy empathy	Emotional resonance	Personal distress
Total secondary trauma score	0.247#	0.332#	0.450#	−0.219	0.488#
Cognitive response	0.297#	0.327#	0.441#	−0.068	0.442#
Emotional response	0.200*	0.265#	0.394#	−0.223*	0.444#
Behavioral response	0.251#	0.286#	0.369#	−0.119	0.526#
Life beliefs	0.135	0.297#	0.356#	−0.170	0.270#
Physiological response	0.199*	0.266#	0.360#	−0.243#	0.386#

* Indicates *P* < 0.05; #indicates *p* < 0.01.

#### 3.4.2 Relationship analysis of secondary trauma and nursing stress in emergency department nurses

The total score of nursing stress is positively correlated with secondary trauma (*r* = 0.209, *p* < 0.05), indicating that more severe secondary trauma is associated with greater perceived nursing stress. Nursing stress is also positively correlated with cognitive response (*r* = 0.231, *p* < 0.01), emotional response (*r* = 0.191, *p* < 0.05), behavioral response (*r* = 0.140), and physiological response (*r* = 0.099). Perceived stress shows significant positive correlations with the total score of secondary trauma (*r* = 0.311, *p* < 0.01) and all response dimensions, including cognitive (*r* = 0.349, *p* < 0.01), emotional (*r* = 0.289, *p* < 0.01), behavioral (*r* = 0.283, *p* < 0.01), and physiological responses (*r* = 0.221, p < 0.05). Control perception is positively correlated with secondary trauma (*r* = 0.337, *p* < 0.01), and also with all response dimensions (*r* values ranging from 0.238 to 0.269, *p* < 0.01). Emotional response is strongly correlated with secondary trauma (*r* = 0.430, *p* < 0.01), cognitive response (*r* = 0.293, *p* < 0.01), perceived stress (*r* = 0.400, *p* < 0.01), control perception (*r* = 0.323, *p* < 0.01), and physiological response (*r* = 0.289, *p* < 0.01). Behavioral response shows moderate correlations with secondary trauma (*r* = 0.140), and other dimensions (*r* values ranging from 0.238 to 0.323, *p* < 0.01). Life beliefs correlate positively with secondary trauma (*r* = 0.292, *p* < 0.01), emotional response (*r* = 0.522, *p* < 0.01), and other stress-related factors (*r* values ranging from 0.211 to 0.352, *p* < 0.01). Physiological response is significantly correlated with secondary trauma (*r* = 0.099) and other dimensions, especially behavioral and emotional responses (*r* values ranging from 0.221 to 0.323, *p* < 0.01). See Table 8.

**Table 8:** Correlation analysis of secondary trauma and nursing stress in emergency department nurses (*r*-values).

Variable	Total nursing stress score	Perceived stress	Control perception	Emotional response
Total secondary trauma score	0.209*	0.311#	0.337#	0.430#
Cognitive response	0.231#	0.349#	0.239#	0.293#
Emotional response	0.191*	0.289#	0.307#	0.400#
Behavioral response	0.140	0.283#	0.238#	0.323#
Life beliefs	0.292#	0.211*	0.352#	0.522#
Physiological response	0.099	0.221*	0.269#	0.289#

*Indicates *p* < 0.05; #indicates *P* < 0.01.

#### 3.4.3 Analysis of the relationship between secondary traumatization and psychological resilience in emergency nurses

The correlation analysis between secondary traumatization and psychological resilience in emergency nurses is shown in Table 9. The total score of secondary traumatization is negatively correlated with the total score of psychological resilience (*r* = −0.261, *p* < 0.01), indicating that the stronger the psychological resilience of emergency nurses, the lower the likelihood of experiencing secondary traumatization. The total score of secondary traumatization is negatively correlated with cognitive response (*r* = −0.057, *p* > 0.05), emotional response (*r* = −0.035, *p* > 0.05), and behavioral response (*r* = −0.253, *p* < 0.01), and with physiological response (*r* = −0.221, *p* < 0.05). Personal confidence and self-efficacy have no significant correlation with the total score of secondary traumatization (*r* = −0.092, *p* > 0.05), but the life belief dimension is significantly positively correlated with the total score of secondary traumatization (*r* = 0.322, *p* < 0.01), suggesting that nurses with stronger life beliefs may show higher adaptability when facing secondary traumatization. Emotional regulation and self-control are significantly negatively correlated with the total score of secondary traumatization (*r* = −0.211, *p* < 0.05), and the behavioral response (*r* = −0.249, *p* < 0.01) and physiological response (*r* = −0.212, *p* < 0.05) are also negatively correlated with emotional regulation and self-control. This suggests that nurses with stronger emotional regulation skills may exhibit lower stress responses in terms of behavior and physiological reactions when coping with secondary traumatization. Interpersonal relationships and social support are negatively correlated with the total score of secondary traumatization (*r* = −0.097, *p* > 0.05), but in the behavioral response dimension, secondary traumatization is significantly negatively correlated with interpersonal relationships and social support (*r* = −0.194, *p* < 0.05). This indicates that nurses with better interpersonal relationships may exhibit lower behavioral stress responses when facing secondary traumatization. Cognitive and problem-solving abilities are negatively correlated with the total score of secondary traumatization (*r* = −0.157, *p* > 0.05), and in the life belief dimension, cognitive and problem-solving abilities are positively correlated with secondary traumatization (*r* = 0.211, *p* < 0.05), suggesting that nurses with stronger cognitive abilities may demonstrate higher psychological adaptation levels in the life belief dimension. Regarding physiological responses, secondary traumatization is significantly negatively correlated with physiological responses (*r* = −0.221, *p* < 0.05), indicating that nurses with stronger psychological resilience may experience less impact from secondary traumatization in terms of physiological stress responses. See Table 9.

**Table 9:** Correlation analysis between secondary traumatization and psychological resilience in emergency nurses (*r* values).

Variable	Psychological resilience	Personal confidence and self-efficacy	Emotional regulation and self-control	Interpersonal relationships and social support	Cognitive and problem-solving abilities
Total secondary trauma score	−0.261^#^	−0.092	−0.211^*^	−0.097	−0.157
Cognitive response	−0.057	−0.048	−0.047	0.074	−0.024
Emotional response	−0.035	0.034	−0.001	0.109	0.032
Behavioral response	−0.253^#^	−0.128	−0.249^#^	−0.194^*^	−0.132
Life beliefs	0.132	0.322^#^	0.246^#^	0.258^#^	0.211^*^
Physiological response	−0.221^*^	−0.103	−0.212^*^	−0.174	−0.087

* Indicates *P* < 0.05; # indicates *P* < 0.01.

### 3.5 Multiple linear regression analysis of secondary traumatization in emergency nurses

**Figure 2:**
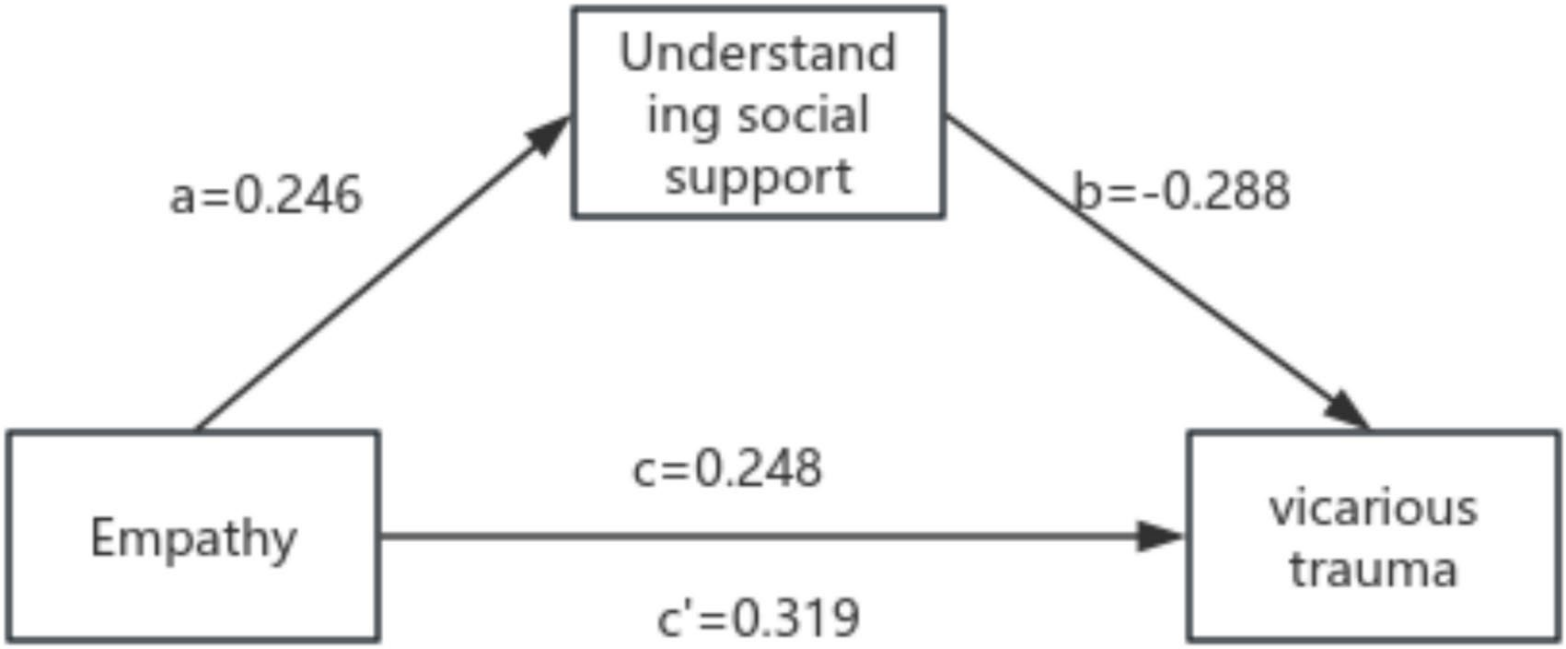
Conceptual diagram of the regression model. This diagram illustrates the relationships between Empathy, Understanding Social Support, and Vicarious Trauma. The arrows represent causal relationships between the variables, with the coefficients (e.g., *a* = 0.246, *b* = −0.288) indicating the regression values for each path.

In this study, the total score of secondary traumatization (STS) in emergency nurses was used as the dependent variable, while independent variables such as personal distress, emotional resonance, fantasy empathy, and exposure to traumatic events were selected. A multiple linear regression analysis was conducted to assess their associations with secondary traumatization. The model fit was satisfactory, with an adjusted *R*^2^ = 0.561, indicating that the regression model explains a substantial portion of the variance in secondary traumatization. Collinearity diagnostics revealed that the tolerance values of the independent variables ranged from 0.61 to 0.99, and the variance inflation factors (VIFs) ranged from 1.01 to 1.38, indicating no issues with multicollinearity. The regression results showed that personal distress, emotional resonance, fantasy empathy, external support, and exposure to traumatic events were significant factors influencing secondary traumatization in emergency nurses. The combined independent variables explained 56.00% of the variance in secondary traumatization. The largest standardized regression coefficient was for personal distress, suggesting that it has the most significant impact on secondary traumatization. See Tables 10, 11 and Figure 2.

**Table 10:** Variable assignment explanation.

Variable	Assignment method	Assignment method
Work experience	1–3 years	1–3 years = 0 (reference)
	4–5 years	1–3 years = 0, 4–5 years = 1, 6–10 years = 0, 11–15 years = 0, 16 + years = 0
	6–10 years	1–3 years = 0, 4–5 years = 0, 6–10 years = 1, 11–15 years = 0, 16 + years = 0
	11–15 years	1–3 years = 0, 4–5 years = 0, 6–10 years = 0, 11–15 years = 1, 16 + years = 0
	16 + years	1–3 years = 0, 4–5 years = 0, 6–10 years = 0, 11–15 years = 0, 16 + years = 1
Personality type	Introverted	Introverted = 0 (reference)
	Extroverted	Introverted = 0, Extroverted = 1, Ambivert = 0
	Ambivert	Introverted = 0, Extroverted = 0, Ambivert = 1
Gender	Male = 0, Female = 1	
Traumatic events	Yes = 0, No = 1	
Psychological trauma training	Yes = 0, No = 1	
Career re-selection	Yes = 0, No = 1	
Empathy total score	Enter original value	
Perspective taking	Enter original value	
Fantasy empathy	Enter original value	
Emotional resonance	Enter original value	
Personal distress	Enter original value	
Total nursing stress score	Enter original value	
Stress perception	Enter original value	
Control sense	Enter original value	
Emotional response	Enter original value	

**Table 11:** Multiple linear regression analysis of secondary traumatization in emergency nurses.

Independent variable	Unstandardized coefficient		Standardized coefficient	*t*-value	*P*-value	95%CI
	*B*	Standard error	*β*			
Constant	87.648	14.889	–	5.887	0.000	(58.092,117.204)
Personal distress	2.242	0.607	0.398	3.694	0.000	(1.040, 3.444)
Emotional resonance	−1.828	0.574	−0.285	−3.181	0.002	(−2.961, −0.695)
Fantasy empathy	−1.766	0.757	0.256	2.484	0.015	(−3.257, −0.275)
Traumatic events	−9.129	3.580	−0.162	−2,550	0.012	(−15.271, −3.310)

## 4 Discussion

### 4.1 The mechanisms of nursing stress, empathy, and psychological resilience on STS

Emergency department (ED) nurses work in high-pressure environments where nursing stress, empathy, and psychological resilience play significant roles in the development of secondary traumatic stress (STS). Nursing stress exacerbates STS by intensifying emotional and cognitive reactions when nurses are exposed to patient trauma, due to high work demands, difficulties in emotional regulation, and cognitive resource depletion. Empathy, particularly emotional resonance and cognitive empathy, shapes the depth of nurses’ perception of patients’ suffering. This perception, through emotional contagion, increases emotional burdens, leading to emotional dysregulation and STS (5, 16–18). Psychological resilience, which is the ability to adapt and recover, helps reduce the impact of nursing stress and excessive empathy on STS by enhancing emotional regulation, cognitive restructuring, and coping strategies. Specifically, nursing stress triggers the stress response system, causing anxiety, depression, and emotional symptoms that deplete psychological resources and reduce adaptability, thereby intensifying the internalization of patients’ traumatic experiences (5, 19, 20). Additionally, nursing stress reduces perceived social support and personal control, which further elevates the risk of STS. Empathy, particularly its emotional and cognitive dimensions, is positively correlated with STS. In high-empathy situations, nurses may experience emotional overinvestment, leading to exhaustion and cognitive overload, exacerbating STS, especially in the ED where acute traumatic events significantly affect emotional and cognitive states. Strong emotional resonance can lead to empathy fatigue, and excessive emotional involvement can trigger psychological health issues. Meanwhile, psychological resilience acts as a protective factor, improving emotional regulation and coping strategies, which enhances adaptability and recovery from trauma, mitigating the negative effects of stress and excessive empathy (21, 22). Studies have shown that individuals with higher resilience tend to employ more effective emotional regulation and problem-solving strategies, which helps them avoid emotional overinvestment and reduces the risk of STS. Therefore, nursing stress, empathy, and psychological resilience interact and influence emotional regulation, cognitive load, and psychological adaptation, ultimately shaping the mechanisms of STS. To address this, interventions aimed at enhancing nurses’ psychological resilience, strengthening emotional regulation skills, and managing work stress effectively are critical in reducing STS among ED nurses.

### 4.2 Clinical significance and application value of the model

The multiple linear regression model of STS in ED nurses developed in this study, based on nursing stress, empathy, and psychological resilience, provides a new assessment tool for clinical practice. It effectively reveals the mechanisms of how these variables impact STS. This model not only has significant predictive efficacy, identifying high-risk individuals, but also provides a theoretical basis for developing intervention strategies, optimizing nursing management, and improving nurses’ psychological health. Firstly, the model can accurately identify individuals at risk for STS under specific circumstances, especially those with strong empathy, high emotional resonance, and low psychological resilience. Through multiple regression analysis, this study verified the significant roles of personal distress, emotional resonance, fantasy empathy, and exposure to traumatic events in STS, particularly in the high-pressure work environment of the ED. The psychological state, emotional load, and emotional regulation ability of nurses significantly impact the occurrence of STS. Based on these findings, hospital administrators can use the model’s risk factors to conduct regular psychological assessments of nurses and intervene early, implementing precise preventive measures (23, 24). Secondly, the clinical application value of the model lies in its ability to provide a basis for developing personalized intervention programs. Hospital managers can interpret the model’s analysis results to design interventions tailored to specific nurse groups. For example, for nurses with strong emotional resonance and low empathetic concern, hospitals can use emotional regulation training, psychological resilience enhancement, and stress management to improve their ability to cope with traumatic events and reduce emotional burdens, thereby decreasing the likelihood of STS (25–28). At the same time, the model’s results offer targeted points for mental health interventions for nursing staff, particularly for those lacking psychological resilience. Emphasis should be placed on enhancing psychological resilience and emotional regulation abilities through systematic psychological support and emotional management programs, effectively increasing their ability to resist psychological stress and trauma and reducing mental health risks (29). Furthermore, based on the results of this study’s model, hospitals can optimize the work environment and management strategies to reduce unnecessary emotional burdens and psychological stress. For example, by optimizing work schedules, strengthening team support, providing regular psychological counseling, and offering emotional support, nurses’ job satisfaction and emotional needs can be improved, reducing emotional exhaustion and burnout caused by high-intensity work, thus lowering the occurrence of STS (30). Lastly, the application of this model provides scientific evidence for the long-term sustainable development of nursing staff’s occupational health. ED nurses in high-pressure and emotionally demanding environments are prone to emotional regulation imbalances and insufficient psychological endurance. The clinical application of this model can enhance systematic assessments of nurses’ psychological health, detect potential risks of emotional exhaustion and psychological crises, and promote the construction of a nursing management model centered on psychological health. Additionally, this model is not limited to the ED; it has the potential for cross-departmental application, offering insights for other nursing staff in high-pressure work environments, fostering the establishment of broader mental health intervention and support systems.

Based on the research findings, we propose a series of specific and actionable interventions for STS among emergency department nurses. First, regarding the two dimensions of empathy—perspective adoption and emotional resonance—we recommend developing dedicated training modules. Through methods such as reflective exercises and role-playing, nurses can improve their perspective adoption skills, enabling them to better understand patients’ emotions and needs, thereby reducing emotional fatigue. Simultaneously, regarding emotional resonance, we recommend improving nurses’ coping abilities through psychological resilience training, teaching them how to manage and regulate their emotional responses to traumatic events to avoid emotional overload. Second, since personal distress and work stress are the main contributing factors to STS, we recommend providing emergency department nurses with stress management courses, such as mindfulness meditation and cognitive behavioral therapy (CBT), to help them effectively manage their emotions and psychological burden under high-pressure environments. Considering the impact of the work environment on mental health, reasonable shift scheduling is also crucial; we recommend reducing long shifts, avoiding excessive workloads, and providing more rest time to help nurses recover their energy and reduce stress. Furthermore, peer support groups and regular mental health assessments are effective interventions. Regular mental health checkups and counseling services can help identify and address the emotional problems of high-risk nurses in a timely manner. Implementing these interventions not only helps reduce STS but also improves nurses’ psychological resilience and overall quality of care. Therefore, future research should further explore the long-term effects of these interventions and promote best practices for mental health management in the emergency department setting.

### 4.3 Innovation and limitations

This study has certain innovative aspects. Firstly, in terms of research design, it combines the work characteristics of emergency department (ED) nurses and considers multiple variables, such as nursing stress, empathy, and psychological resilience, through multiple linear regression analysis to explore the mechanisms by which these factors influence STS. This research fills a gap in the existing literature regarding the multidimensional study of STS in ED nurses. Secondly, this study is the first to introduce psychological resilience as a variable, exploring its role in alleviating STS, particularly in terms of its moderating effect on work stress and emotional impact. This provides new ideas and perspectives for clinical nursing interventions. Additionally, the study investigates the relationship between empathy and STS, and through detailed analysis of the dimensions of empathy, it further reveals how psychological factors such as emotional resonance and fantasy empathy influence the occurrence of STS through emotional regulation and cognitive responses. Through regression analysis, this research also explores the significant roles of personal distress, empathetic concern, imagination, and exposure to traumatic events in the development of STS, constructing a relatively comprehensive theoretical framework. This provides a new reference for research in related fields. Although this study offers certain innovation in exploring the mechanisms and constructing models for STS in ED nurses, it still faces limitations related to sample sources, research design, and measurement tools. Future research should improve research methods based on broader groups and longer time dimensions to explore the multidimensional influencing factors and their interactive mechanisms of STS. This will provide theoretical evidence and practical guidance for further optimizing nursing intervention strategies and improving nurses’ mental health.

In this study, we used convenience sampling, and the sample was drawn from only one hospital, which limited the external validity and generalizability of the results. The findings may not be fully applicable to other emergency department nurse groups or hospitals in different regions, and should therefore be interpreted with caution. Secondly, this study employed a cross-sectional design, meaning we can only reveal associations between variables, not causal relationships. Since the data were collected at a single time point, future longitudinal studies could better explore causal relationships between variables and reveal the directionality of associations. Furthermore, all measurements were self-reported, which may have led to response bias, where participants may have answered based on social expectations or underestimated the degree of emotional distress and secondary traumatic stress. Future research could consider using objective scales or multi-source data to reduce the impact of such bias. Finally, given that all variables in this study came from the same data source, common method bias (CMV) may exist, which could exaggerate the relationships between variables. While we did not specifically control for CMV, this potential issue has been discussed in the limitations section, and future research could mitigate this effect by using multiple information sources or multiple methods of data collection.

## 5 Conclusion

This study examines the associations between nursing stress, empathy, psychological resilience, and STS among emergency department nurses. By developing a comprehensive model, we assess how these variables are collectively associated with STS. The results indicate that these factors significantly influence the occurrence of STS through complex relationships. Increased nursing stress, heightened empathy, and reduced psychological resilience are associated with a higher susceptibility to STS. Key mediators include personal distress, emotional resonance, and prior trauma exposure. Multiple regression analysis revealed that nursing stress, empathy traits, resilience levels, and personal trauma history are important factors associated with the severity of STS.

## Data Availability

The original contributions presented in the study are included in the article/supplementary material, further inquiries can be directed to the corresponding author/s.
